# Multicenter retrospective cohort study of the association between surgery for odontoid fractures in the elderly and in-hospital outcomes

**DOI:** 10.1038/s41598-023-33158-3

**Published:** 2023-04-18

**Authors:** Zamir Merali, Peng F. Zhang, Rachael H. Jaffe, Blessing N. R. Jaja, Erin M. Harrington, Armaan K. Malhotra, Christopher W. Smith, Yingshi He, Michael Balas, Andrew S. Jack, Michael G. Fehlings, Jefferson R. Wilson, Christopher D. Witiw

**Affiliations:** 1 Department of Surgery, Division of Neurosurgery, University of Toronto, Toronto, M5T1P5 Canada; 2 Department of Surgery, Division of Neurosurgery, St. Michael’s Hospital, Toronto, M5B1W8 Canada; 3 Institute of Health Policy Management and Evaluation, University of Toronto, Toronto, M5T1P8 Canada; 4 St. Michael’s Hospital, Li Ka Shing Knowledge Institute, Toronto, M5B1T8 Canada; 5 Division of Neurosurgery, University of Alberta, Edmonton, T6G1Z1 Canada; 6 Department of Surgery, Division of Neurosurgery, Toronto Western Hospital, Toronto, M5T2S8 Canada

**Keywords:** Trauma, Fracture repair, Geriatrics, Prognosis, Risk factors

## Abstract

Odontoid fractures are increasingly prevalent in older adults and associated with high morbidity and mortality. Optimal management remains controversial. Our study aims to investigate the association between surgical management of odontoid fractures and in-hospital mortality in a multi-center geriatric cohort. We identified patients 65 years or older with C2 odontoid fractures from the Trauma Quality Improvement Program database. The primary study outcome was in-hospital mortality. Secondary outcomes were in-hospital complications and hospital length of stay. Generalized estimating equation models were used to compare outcomes between operative and non-operative cohorts. Among the 13,218 eligible patients, 1100 (8.3%) were treated surgically. The risk of in-hospital mortality did not differ between surgical and non-surgical groups, after patient and hospital-level adjustment (OR: 0.94, 95%CI: 0.55–1.60). The risks of major complications and immobility-related complications were higher in the operative cohort (adjusted OR: 2.12, 95%CI: 1.53–2.94; and OR: 2.24, 95%CI: 1.38–3.63, respectively). Patients undergoing surgery had extended in-hospital length of stay compared to the non-operative group (9 days, IQR: 6–12 days vs. 4 days, IQR: 3–7 days). These findings were supported by secondary analyses that considered between-center differences in rates of surgery. Among geriatric patients with odontoid fractures surgical management was associated with similar in-hospital mortality, but higher in-hospital complication rates compared to non-operative management. Surgical management of geriatric patients with odontoid fractures requires careful patient selection and consideration of pre-existing comorbidities.

## Introduction

Fractures of the C2 odontoid process are the most common isolated spine fracture in geriatric patients and are associated with high morbidity and mortality^1–4^. The number of individuals over 65 years of age in the United States is expected to increase faster than any other age group to over 70 million in the next decade^5^. Given the rapid expansion of this population and this high incidence of odontoid fractures, geriatric spine fractures have been described as an emerging health crisis^6^.

Despite the increasing frequency of these fractures there remains uncertainty about the optimal management strategy^7^. Non-operative treatment is associated with a risk of fibrous non-union and high 1-year mortality^8–13^. Surgery, however, is associated with high peri-operative risks given the prevalence of poor bone health and medical comorbidities in an elderly population^14–16^. Previous retrospective studies have found surgical treatment of geriatric odontoid fractures to be associated with lower mortality at 30 days when compared to non-operative treatment^17–20^. A prospective study comparing surgical fixation with non-operative treatment found a trend toward lower mortality in patients treated surgically^21^. In contrast others have advocated that non-operative management is an acceptable treatment option despite lower rates of osseous union^11,18^. While most previous studies have used data gathered at a single institution, few have examined a multi-center cohort^4,17^.

In this study we conducted a retrospective analysis using data from the American College of Surgeons (ACS) Trauma Quality Improvement Program (TQIP). The objective of this study was to determine the association between surgical management of odontoid fractures and in-hospital mortality and complications in a large multi-center cohort of geriatric patients.

## Methods

### Research ethics board approval

The study design was a multicenter retrospective cohort study. This study number 20–338 was approved by the Unity Health Toronto Research Ethics Board (Toronto, Ontario, Canada) in February of 2021. Study procedures were followed in accordance with the ethical standards of the institutional committee on human experimentation and with the Helsinki Declaration of 1975. This study used only de-identified retrospective patient data, and individual participant informed consent was waived by the Unity Health Toronto Research Ethics Board.

### Data source

We analyzed data from the ACS TQIP database from 2010 to 2018. The TQIP combines data from over 450 ACS and state verified Level 1 and 2 trauma centers across North America. Hospital and patient-level variables are collected for each included patient. The dataset excludes patients without signs of life on arrival to hospital, patients with pre-existing advanced directive to limit life sustaining interventions, patients who were discharged from the emergency department (ED), or patients who had severe burns. The dataset undergoes auditing procedures and internal and external validity checks.

### Study eligibility

Patients aged ≥ 65 years with a diagnosis of fracture of the C2 odontoid process were identified using the Abbreviated Injury Scale (AIS) pre-dot code 650228. We excluded patients with non-survivable injury (AIS score of 6 in any body region), non-blunt mechanism of injury, and with severe injuries (AIS score of ≥ 3) in any non-spine body region. In addition, we excluded patients with spinal cord injury by excluding patients with another AIS score in the spine region of ≥ 3.

### Patient and hospital-level characteristics

We collected patient demographic characteristics including age, sex, race, insurance type, and comorbidities. Patient frailty was evaluated by a 5-factor modified Frailty Index (mFI-5)^23^. We collected injury characteristics such as mechanism of injury, AIS score for each body region, total Injury Severity Score (ISS), the need for mechanical ventilation in the ED, shock (systolic blood pressure (SBP) < 90mm Hg) upon arrival to the ED, Glasgow Coma Scale (GCS) score on arrival, and alcohol and drug tests^24^. Hospital characteristics included trauma center level, number of adult beds, teaching status, year of injury, and the number of C2 odontoid fractures seen at the facility.

### Exposure

Surgical procedures for cervical fusion were identified using the International Classification of Diseases, 9th/10th revision, procedure classification system (ICD-9-PCS and ICD-10-PCS) codes (Supplemental Table S1). Two patient cohorts were defined, those who underwent surgical fixation during hospitalization and those who did not.

### Outcomes

The primary outcome was in-hospital mortality. Secondary outcomes included major in-hospital complication, immobility-related complications, extended hospital length of stay (LOS) and discharge destination (dichotomized as routine versus non-routine). Major complication (defined as primary complications in the analysis) was a composite outcome defined in the TQIP dataset as the occurrence of one or more of the following: acute kidney injury, cardiac arrest (with cardiopulmonary resuscitation), acute respiratory distress syndrome (ARDS), decubitus ulcer, surgical site infection, myocardial infarction, pneumonia, ventilator-associated pneumonia, pulmonary embolism, stroke, catheter-related bloodstream infection, unplanned return to the operating room, unplanned admission to the intensive care unit, or severe sepsis. Immobility-related complication was a composite outcome consisting of one or more of the following: decubitus ulcer, pneumonia, or pulmonary embolism. Subjects lost to follow-up or with missing outcome data were assumed to be missing completely at random.

### Statistical analysis

The baseline characteristics of the cohort were reported as counts with percentages for categorical variables. Hospital length of stay (LOS) and the proportion of patients enrolled and undergoing surgery at each facility were found to have positively skewed distributions and were thus presented as median with inter-quartile ranges (IQRs). In accordance with the STROBE recommendations we used Standardized Mean Differences (SMDs) to compare the baseline characteristics of the cohorts. We compared the outcomes between the surgically and non-surgically treated patients using a generalized estimating equation (GEE) model with facility used as a clustering identifier and an exchangeable working correlation structure^25,26^. For binary outcomes, we assumed a binomial distribution and specified a logit link function. For hospital LOS, we assumed a gaussian distribution. Covariates included in the model were year of admission, hospital characteristics (teaching status, trauma center level, case load), patient demographics (age, race, gender, insurance status), and clinical characteristics (GCS, injury mechanism, SBP < 90 in ED, ventilator during admission, Charlson Comorbidity Index, mFI5, AIS score in each body region, ISS).

We performed several secondary analyses. First, we included interaction terms in adjusted models to examine whether the effect of surgery varies with age, LOS and frailty. Next, we restricted the analysis to centers including at least 5 patients for three years of participation in TQIP. In a final analysis, we used a random effects (mixed-effects) logistic regression models to investigate whether there are substantial between-center differences in the outcomes that could not be explained by random variation or the patient and hospital characteristics included in the models. To ensure a family-wise type-1 error rate less than 0.05 in our primary and secondary analyses we used a Bonferroni correction, setting the significance cut-off at *p* < 0125.

## Results

### Study population

**Figure 1:**
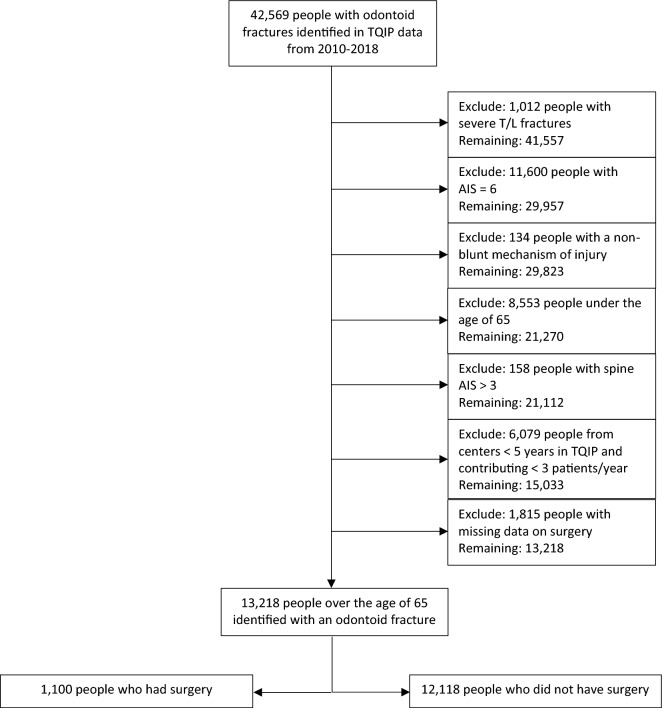
Flow diagram depicting criteria for inclusion in the analytic cohort. Analytical data excludes patients from centers contributing one patient or less. *AIS* Abbreviated injury scale, *TQIP* Trauma quality improvement program, *T/L* Thoracolumbar. This figure is original to this submission so no credit or license is needed.

A total of 13,218 patients treated at 678 trauma centers met eligibility criteria (Fig. 1). The number of patients who underwent surgery was 1,100 (8.3%), and the number who were treated non-operatively was 12,118 (91.7%). On average, patients who underwent surgery were younger and had a lower comorbidity burden (Table 1). Patients who did and did not have surgery were comparable with respect to sex distribution, ISS, mechanism of injury, rates of assisted ventilation in the ED and hemorrhagic shock in the ED (Table 1). The most common mechanism of injury in both groups was fall, followed by motor vehicle collision. The distribution of in-hospital complications in the cohort is shown in (Supplemental Table S2).

**Table 1:** Summary characteristics of the analytic cohort.

	No Surgery	Surgery	Total	SMD
Demographic characteristics				
Age—n (%)				0.393
65–69	925 (7.7)	127 (11.9)	1052 (8.1)	
70–79	3463 (29.1)	457 (42.8)	3920 (30.2)	
80–89	5945 (49.9)	442 (41.4)	6387 (49.2)	
90 +	1581 (13.3)	43 (4.0)	1624 (12.3)	
Male—n (%)	5057 (41.7)	527 (47.9)	5584 (42.3)	0.125
Race—n (%)				0.021
White	11,114 (93.0)	983 (91.6)	12,097 (92.9)	
Asian	335 (2.8)	48 (4.5)	383 (2.9)	
Black	398 (3.3)	34 (3.2)	432 (3.2)	
Other	103 (0.9)	8 (0.8)	111 (0.9)	
Insurance—n (%)				0.028
Government	6216 (80.9)	521 (80.0)	6737 (80.8)	
Other	1009(13.1)	87 (13.4)	1096 (13.2)	
Private	456 (5.9)	43 (6.6)	449 (6.0)	
Frailty Index—n (%)				0.008
0	2277 (21.7)	195 (21.0)	2472 (21.7)	
1	4550 (43.5)	414 (44.5)	4964 (43.5)	
= > 2	3645 (34.8)	322 (34.6)	3967 (34.8)	
Injury/presentation characteristics				
ISS ≥ 16—n (%)	406 (3.4)	22 (2.0)	428 (3.2)	0.076
GCS—n (%)				0.132
3–8	155 (1.5)	9 (1)	164 (1.4)	
9–12	181 (1.7)	9 (1.0)	190 (1.7)	
13–14	1308 (12.5)	80 (8.6)	1388 (12.2)	
15	8828 (84.3)	833 (89.5)	9661 (84.7)	
Mechanism of Injury—n (%)				0.012
MVC	1396 (11.5)	122 (11.1)	1518 (11.5)	
Fall	10,449 (86.2)	948 (86.2)	11,397 (86.2)	
Others	273 (2.3)	30 (2.7)	303 (2.3)	
Assisted ventilation in ED—n (%)	260 (2.2)	20 (1.9)	280 (2.2)	0.022
Hemorrhagic Shock—n (%)	96 (0.8)	3 (0.3)	99 (0.7)	0.060
Transferred from another institution—n (%)	6947 (63.2)	708 (70.3)	7655 (63.8)	0.148
Positive alcohol test—n (%)	284 (2.4)	41 (3.8)	325 (2.5)	0.091
Positive drug test—n (%)	880 (7.3)	92 (8.4)	972 (7.4)	0.042
CCI—n (%)				0.281
2	662 (5.5)	104 (9.5)	766 (5.8)	
3	2215 (18.3)	300 (27.3)	2515 (19.0)	
4	8119 (67.0)	618 (56.2)	8717 (66.1 )	
= > 5	1122 (9.3)	78 (7.1)	1200 (9.1)	
Hospital characteristics				
Level I trauma center—n (%)	5531 (45.6)	464 (42.2)	5995 (45.4)	0.069
Teaching Status—n (%)				0.034
Community	5353 (44.2)	525 (47.8)	5878 (44.5)	
Non-teaching	1730 (14.3)	113 (10.3)	1843 (14.0)	
University	5020 (41.5)	460 (41.9)	5480 (41.5)	

*SMD* standardized mean difference, *ISS* Injury severity score, *GCS* Glasgow coma scale, *MVC* Motor vehicle collision, *ED* Emergency department, *CCI* Charlson comorbidity index.

### Effect of surgery on outcomes

Estimates of the association between surgery and the outcomes are as shown in Table 2. We found fewer patients who had surgery died in-hospital (N = 32, 2.9%), compared with those who were treated non-operatively (N = 430, 3.6%). In an adjusted analysis no differences were noted in the risk of in-hospital mortality between the two groups (OR: 0.94, 95%CI: 0.55–1.60) (Supplemental Table S3). The proportion of patients who had primary complications was higher among those who underwent surgery compared with those treated non-operatively (N = 114, 10.4% versus N = 500, 4.1%; *p* < .001). In unadjusted and adjusted models, the risk of primary complications was higher in the surgical group compared with the non-operative group (adjusted OR: 2.12, 95%CI: 1.53–2.94). Similarly, the risk of immobility-related complications was higher in the surgical group than the group treated non-operatively (adjusted OR: 2.24, 95%CI: 1.38–3.63). The patients undergoing surgery spent approximately 5.5 days longer in hospital than those treated non-operatively, after accounting for covariates (5.51, 95%CI: 4.22–6.78, *p* < .0001).

**Table 2:** Association of surgery with outcomes in geriatric C2 fractures.

	No surgery (%)	Surgery (%)	Unadjusted OR (95% CI)	Adjusted OR (95% CI)
All centers (N = 13,218)	n = 12,118	n = 1100		
Mortality in-hospital	430 (3.6)	32 (2.9)	0.79 (0.54–1.17)	0.94 (0.55–1.60)
Primary complication	500(4.1)	114 (10.4)	2.73 (2.22–3.35)	2.12 (1.53–2.94)
Immobility complication	208 (1.7)	39 (3.6)	2.11 (1.39–3.22)	2.24 (1.38–3.63)
Routine discharge	6997 (65.0)	709 (75.8)	1.76 (1.49–2.08)	2.38 (1.92–2.97)
High vol. centers (N = 10,608)				
Mortality In-hospital	370 (3.8)	29 (3.3)	0.85 (0.56–1.29)	0.95 (0.53–1.72)
Primary complications	422 (4.3)	98 (11.2)	2.80 (2.23–3.50)	2.17 (1.53–3.10)
Immobility complications	181 (1.9	37 (4.2)	2.31 (1.50–3.57)	2.24 (1.35–3.73)
Routine discharge	5672 (64.1)	554 (73.2)	1.60 (1.35–1.90)	2.15 (1.72–2.68)

*OR* Odds ratio, *CI* Confidence interval.

### Secondary analyses

#### Interactions

We noted an interaction of surgery with age for immobility-related complications (*p* = .002), and surgery with frailty index for primary complications (*p* = .007). Test of other interactions were not significant.

#### High volume centers

There were 248 (36.6%) facilities enrolling at least 5 patients for at least 3 years of participation in TQIP (N = 10,608) (Table 2). The risk of in-hospital mortality did not statistically differ between surgically treated and non-surgically treated groups (OR: 0.94, 95%CI:0.55–1.60). The risk of primary and immobility-related complications was twice higher among the surgically treated group compared to the non-surgically treated group. The surgical group also spent on average 4.6 days longer in hospital than the latter group (4.59, 95%CI: 3.98–5.20).

#### Between-center differences

The number of patients enrolled varied between 1 And 142 patients per facility (median, 41; IQR: 23, 75). The proportion of patients that had surgery varied between 0(0%)–32 (100%) per facility (median, 2 (6.3%); IQR:1 (1.9%), 6 (12.1%)) (Supplemental Table S4). In fully adjusted analysis (Supplemental Table S5), we noted no difference in mortality risk on comparing facilities in the highest quartile for surgery for C2 fractures to facilities that treated all patients non-operatively (adjusted OR, 1.07, 95% CI: 0.71–1.60). After accounting for demographic, clinical and hospital covariates, we noted no difference in primary complication or immobility-related complication on comparing patients from facilities treating most patients surgically to their counterparts from facilities treating all patients non-operatively (adjusted OR for primary complications, 0.95, 95%CI:0.65–1.39; immobility OR, 1.06, 95%CI:0.61–1.82). Between center differences explained 4.4% of the variance in mortality, 8.1% of the variance in primary complications, 17.0% of the variance in immobility-related complications, and 15.5% of the variance in LOS.

## Discussion

This study assesses the effect of surgery on in-hospital mortality and complications for a multi-center cohort of 13,218 geriatric patients with odontoid fractures. Patients treated surgically had similar in-hospital mortality as patients treated non-operatively after controlling for patient and hospital characteristics. Surgical management, however, was associated with higher rates of in-hospital complications.

Our results are comparable to previous studies that have examined mortality among geriatric patients with odontoid fractures. White et al. performed a meta-analysis of geriatric patients treated surgically for type 2 odontoid fractures and reported an in-hospital mortality rate of 6.2%^27^. Chen et al. reported a 30-day mortality of 3.6% among geriatric patients treated surgically for type 2 odontoid fractures^28^. The study by Chapman et al. reported 30-day mortality of 7% among geriatric patients with odontoid fractures treated surgically^17^. We found 2.9% mortality among patients treated with surgery, which is comparable to previous reports. In our dataset 8.3% of patients underwent surgery, which is a lower incidence than that reported by other studies that have made use of registry data. The study by Robinson et al. reported an incidence of surgery of 22% in a cohort of patients aged ≥ 70 years^29^. In contrast the study by Dhall et al. reported an incidence of surgery of 10.3% in a cohort of octogenarians^4^. Our study included data from a broad range of Level 1 and 2 trauma hospitals across North America and the lower rate of surgery in our cohort may represent differing practice patterns. In addition, we included patients in our cohort based on the AIS predot code, which is not specific for type 2 odontoid fractures but includes all odontoid fractures.

Several studies comparing surgical and non-operative management of patients with odontoid fractures have been reported. One study retrospectively reviewed 322 geriatric patients with odontoid fractures and found that surgically treated patients had a trend towards lower mortality at 1-year follow up when compared to patients treated non-operatively, but this finding was not statistically significant^17^. Vaccaro et al. prospectively followed 159 geriatric patients treated surgically or non-operatively and reported better functional outcomes and a trend towards lower mortality in the surgically treated patients at 1-year follow up but again, the reduction in mortality failed to meet statistical significance^21^. A multi-center retrospective study by Robinson et al. examined 3,375 elderly patients with C2 fractures and reported lower 1-year mortality among patients treated surgically when adjusting for covariates^29^. A systematic review by Robinson et al. included 1,284 cases of geriatric patients with odontoid fractures and found lower mortality among patients treated surgically^30^. We found no significant difference in in-hospital mortality between patients that were treated surgically and those treated non-operatively after adjusting for covariates. In addition, we conducted a secondary between-center analysis that confirmed these findings. It is important to note that these studies examined mortality at 1-year follow up, while we used in-hospital mortality as an outcome.

Few studies have reported the association between surgical management of geriatric odontoid fractures and in-hospital complications. Vaccaro et al. reported similar rates of complications in surgically treated and non-operative patients^21^. Similarly, the systematic review by Robinson et al. reported similar complication rates between surgically treated and non-operative patients but noted that only a small number of studies in their analysis reported complications rates^30^. The retrospective analysis by Dhall et al. examined octogenarians with odontoid fractures and found higher rates of in-hospital complications such as pneumonia, decubitus ulcer, and ARDS among patients treated with surgery compared to non-operative management^4^. In our cohort the frequency of all complications and immobility-related complications were significantly higher in patients treated surgically when adjusting for covariates. These results suggest that while in-hospital complication rates are higher among surgically treated patients the absolute rate of complications is acceptable. Surgical decision making should thus consider the increased risk of complications with potential benefits and patient comorbidities. In addition, the interactions we found between surgery and age/frailty suggests that extra care should be taken when offering surgery to patients with higher age or greater frailty.

Given this study’s observational nature, we are limited in controlling for baseline differences in the surgical and non-operative cohort. Although we made use of a multi-variate GEE model in our primary analysis, there may be unobserved imbalances between the cohorts that we cannot control for. For example, we were not able to control for fracture morphology on imaging studies, which can affect surgical decision making and success of treatment^31^. Our dataset included data on in-hospital mortality and complications, but we were not able to assess patient outcomes after hospital discharge. We were not able to compare outcomes at a longer-term follow up period, which may have been more clinically meaningful^7,32,33^. Our dataset did not permit comparison of patient functional status and we were not able to assess the effect of surgical management on these outcomes. Lastly, we were not able to compare rates of non-union between patients treated surgically and non-operatively.

To our knowledge, this is the largest study to compare outcomes between geriatric patients with odontoid fractures treated surgically and non-operatively. In-hospital mortality did not differ between patients treated surgically and those treated non-operatively. In-hospital complications, however, were higher among patients treated surgically, especially in patients with higher age or frailty. These results emphasize that surgical management of geriatric patients with odontoid fractures is appropriate for certain patients but requires careful patient selection and consideration of pre-existing comorbidities. Future studies may attempt to examine outcomes at a longer follow up period and better understand specific patient level factors that affect outcomes in this population.

## Supplementary Information

Supplementary Information.

## Data Availability

The data that support the findings of this study are available from American College of Surgeons (ACS) Trauma Quality Improvement Program (TQIP) but restrictions apply to the availability of these data, which were used under license for the current study. Data from this study is owned by the ACS and it is publicly available, but a request has to be made to the ACS. The corresponding author of this paper can be contacted for guidance in requesting access to this data.
