# Supplementary material for: Multicenter retrospective cohort study of the association between surgery for odontoid fractures in the elderly and in-hospital outcomes
Source: Sci Rep. 2023 Apr 18;13:6276. doi: 10.1038/s41598-023-33158-3 (PMC10113203; doi:10.1038/s41598-023-33158-3)
Supplement: Supplementary file 1 — Supplementary Information. [file 41598_2023_33158_MOESM1_ESM.pdf]

Multicenter Retrospective Cohort Study of the Association between Surgery for Odontoid Fractures in the Elderly and in-hospital Outcomes

Zamir Merali, MD, MSc, Peng F. Zhang, BSc, Rachael H. Jaffe, BSc, Blessing N. R. Jaja, MD, PhD, Erin M. Harrington, BMSc, Armaan K. Malhotra, MD, Christopher W. Smith, PhD, Yingshi He, MSc, Michael Balas, BHSc, Andrew S. Jack, MD, MSc, Michael G. Fehlings, MD, PhD, Jefferson R. Wilson, MD, PhD, Christopher D. Witiw, MD, MSc

Complication	Percent with complication	
	Surgery	Non-Operative
Pneumonia	3.93	2.06
Unplanned admission to the ICU	2.01	0.69
Decubitus ulcer	1.31	0.65
Pulmonary embolism	1.14	0.20
Acute kidney injury	0.87	0.58
Unplanned return to the OR	0.87	0.04
ARDS	0.79	0.33
Stroke / CVA	0.79	0.28
Cardiac arrest with CPR	0.61	0.53
Severe sepsis	0.61	0.39
Myocardial infarction	0.26	0.51
Deep surgical site infection	0.09	0.02
VAP	0.09	0.08
Organ/space surgical site infection	0.00	0.07
Systemic sepsis	0.00	0.03
Catheter-Related Blood Stream Infection	0.00	0.06
CLABSI	0.00	0.00

Abbreviations: ICU, intensive care unit; OR, operating room; ARDS, acute respiratory distress syndrome; CVA, cerebrovascular accident; CPR, cardiopulmonary resuscitation; VAP, ventilator-associated pneumonia; CLABSI, central line-associated bloodstream infection.

Supplementary Table S1. Frequency of complications in the surgical and non-surgical cohorts.

died	Odds ratio	Robust standard error	z	P> |z|	[95% confidence interval]	
1.surg	.9363976	.2560274	-0.24	0.810	.5479325	1.600271
aged						
1	1.107991	.5379289	0.21	0.833	.4278371	2.869419
2	1.837497	.8576613	1.30	0.192	.7360777	4.587007
3	2.440638	1.180399	1.84	0.065	.9458559	6.297695
1.female	.5231869	.0606331	-5.59	0.000	.4168785	.656605
racn						
1	.3154915	.1712464	-2.13	0.034	.1088835	.9141413
2	.7409675	.2142436	-1.04	0.300	.4204191	1.305918
3	.5283765	.4212461	-0.80	0.424	.1107456	2.520929
yr						
2011	.9165872	.251699	-0.32	0.751	.5350939	1.570065
2012	.8096268	.2204596	-0.78	0.438	.4747933	1.380591
2013	.9655352	.247185	-0.14	0.891	.5845928	1.594714
2014	.7490278	.18474	-1.17	0.241	.4619106	1.214613
2015	.6303256	.1629003	-1.79	0.074	.3798235	1.046039
2016	.5782059	.1476045	-2.15	0.032	.3505804	.9536245
2017	1	(empty)				
2018	1	(empty)				
insur						
1	.8738835	.1976845	-0.60	0.551	.5609187	1.361467
2	.7255732	.2553417	-0.91	0.362	.3640229	1.446218
hstatus						
1	1.234602	.2865744	0.91	0.364	.7833339	1.945839
2	.930154	.1512786	-0.45	0.656	.676265	1.27936
1.hlev	.7622649	.1232041	-1.68	0.093	.5552993	1.046369
gcs						
1	.1540288	.0580332	-4.96	0.000	.0736031	.3223352
2	.0865044	.028778	-7.36	0.000	.0450675	.1660403
3	.0368808	.0110859	-10.98	0.000	.0204617	.0664752
mech						
1	.4669395	.1178218	-3.02	0.003	.2847599	.7656713
2	.6812347	.3698431	-0.71	0.480	.2350598	1.974309
cci						
3	.9403694	.5979992	-0.10	0.923	.2703976	3.270349
4	1.479105	.9263692	0.62	0.532	.4333993	5.047889
5	1.629149	1.057962	0.75	0.452	.456241	5.817378
findex						
1	1.006686	.1706416	0.04	0.969	.7221182	1.403394
2	1.103393	.1897189	0.57	0.567	.7877233	1.545564
beds						
1	2.735871	1.805359	1.53	0.127	.7505868	9.972186
2	3.101295	2.043557	1.72	0.086	.8524226	11.28317
3	2.73235	1.815717	1.51	0.130	.7428325	10.05036
1.iss	2.485601	.9167438	2.47	0.014	1.206396	5.121216
cload	.9930581	.0027694	-2.50	0.012	.987645	.9985008
Head						
1	1.082258	.1807487	0.47	0.636	.7801373	1.50138

2	.9650369	.2051932	-0.17	0.867	.6361436	1.463972
Face						
1	1.229186	.1617076	1.57	0.117	.9498096	1.590739
2	.8657692	.2494349	-0.50	0.617	.4922257	1.52279
Neck						
1	2.638758	1.282464	2.00	0.046	1.017903	6.840574
2	2.599476	1.070664	2.32	0.020	1.159566	5.827417
Thorax						
1	.776096	.2671854	-0.74	0.462	.3952515	1.523903
2	1.46482	.4777109	1.17	0.242	.7730156	2.77575
Abdomen						
1	2.264884	1.046464	1.77	0.077	.9157098	5.601883
2	1.683103	1.141709	0.77	0.443	.4453591	6.360792
Upper_Extrem						
1	.9795037	.1889797	-0.11	0.915	.6710893	1.429657
2	.89331	.2309311	-0.44	0.663	.538216	1.482681
Lower_Extrem						
1	.8221533	.1755247	-0.92	0.359	.5410364	1.249336
2	1.563139	.4025965	1.73	0.083	.9435503	2.589584
1.edvnt	1.681639	.5655181	1.55	0.122	.8699263	3.250747
1.hemoshk	1.919137	.9898798	1.26	0.206	.6983305	5.27413
_cons	.3595135	.3220215	-1.14	0.253	.0621272	2.080409

Supplementary Table S2. Result of multivariable analysis association between surgery and mortality in-hospital.

Quartile	Percentage of surgically treated patients	No. of centers	No. of patients
1	1.0 – 4.5	51	2632
2	4.6 – 8.2	61	2677
3	8.3 – 13.9	89	2525
4	14.0 – 100%	124	2551

Supplementary Table S3. Distribution of facilities according to quartiles of the proportion of patients treated surgically.

died	Odds ratio	Robust standard error	z	P>|z|	[95% confidence interval]	
surgFRAC_Q						
1	1.255755	.2777402	1.03	0.303	.8140318	1.937173
2	1.000289	.2030237	0.00	0.999	.6719875	1.488983
3	.9953632	.2323283	-0.02	0.984	.6299443	1.572755
4	1.066945	.2206399	0.31	0.754	.7114056	1.600174
aged						
1	1.126554	.5480996	0.24	0.807	.4341295	2.923379
2	1.87108	.8771011	1.34	0.181	.7465806	4.689297
3	2.480938	1.202372	1.87	0.061	.9595909	6.414246
1.female	.5239772	.0607954	-5.57	0.000	.4173979	.6577707
racn						
1	.3219603	.1748789	-2.09	0.037	.111034	.9335741
2	.7635726	.2265029	-0.91	0.363	.426927	1.365674
3	.5158922	.4209555	-0.81	0.417	.1042315	2.5534
yr						
2011	.9260751	.2559865	-0.28	0.781	.5387116	1.591974
2012	.8098124	.2223826	-0.77	0.442	.4727547	1.38718
2013	.9638744	.2475643	-0.14	0.886	.5826334	1.594577
2014	.7429321	.1865267	-1.18	0.237	.454192	1.215231
2015	.6344748	.1648398	-1.75	0.080	.3813009	1.05575
2016	.5826993	.1501555	-2.10	0.036	.3516404	.9655844
2017	1	(empty)				
2018	1	(empty)				
insur						
1	.8759903	.1993578	-0.58	0.561	.5607676	1.368408
2	.7405105	.2574243	-0.86	0.387	.3746514	1.463643
hstatus						
1	1.2505	.2939615	0.95	0.342	.7888373	1.982349
2	.9381479	.1537514	-0.39	0.697	.680408	1.29352
1.hlev	.7531152	.1198625	-1.78	0.075	.5512999	1.028809
gcs						
1	.1551462	.0586707	-4.93	0.000	.0739345	.325563
2	.0876198	.0290441	-7.35	0.000	.0457559	.1677866
3	.03736	.0112184	-10.95	0.000	.02074	.0672982
mech						
1	.4607276	.1175289	-3.04	0.002	.2794524	.7595926
2	.6725266	.3718481	-0.72	0.473	.2275463	1.987692
cci						
3	.927298	.5953339	-0.12	0.906	.2634754	3.263612
4	1.456771	.9202845	0.60	0.551	.4223401	5.024815
5	1.597911	1.051055	0.71	0.476	.4402095	5.800237
findex						
1	1.006736	.1713156	0.04	0.969	.7212191	1.405284
2	1.105491	.1912038	0.58	0.562	.7876496	1.551592
beds						
1	2.689794	1.771592	1.50	0.133	.7397556	9.780248
2	3.027774	1.991766	1.68	0.092	.8340191	10.99185
3	2.733797	1.819781	1.51	0.131	.7415749	10.07807

1.iss	2.469941	.9205917	2.43	0.015	1.189675	5.127962
cload	.992349	.0029415	-2.59	0.010	.9866004	.998131
Head						
1	1.086022	.1821583	0.49	0.623	.7817482	1.508727
2	.9564799	.2049838	-0.21	0.836	.6284263	1.455785
Face						
1	1.225764	.1628989	1.53	0.126	.9446818	1.590479
2	.8647568	.2492602	-0.50	0.614	.4915198	1.521412
Neck						
1	2.738535	1.355576	2.04	0.042	1.037942	7.225427
2	2.681157	1.104834	2.39	0.017	1.195541	6.012844
Thorax						
1	.7804169	.2705342	-0.72	0.474	.3955985	1.539567
2	1.453677	.4803127	1.13	0.258	.7607124	2.777892
Abdomen						
1	2.251713	1.044754	1.75	0.080	.906924	5.590558
2	1.736209	1.182819	0.81	0.418	.4567805	6.599275
Upper_Extrem						
1	.9761944	.1889493	-0.12	0.901	.6680058	1.426568
2	.911346	.2377111	-0.36	0.722	.5465879	1.519521
Lower_Extrem						
1	.8114405	.1745354	-0.97	0.331	.5323153	1.236928
2	1.575918	.4070804	1.76	0.078	.9498543	2.61463
1.edvnt	1.708945	.5692471	1.61	0.108	.8895983	3.282933
1.hemoshk	1.911051	1.005146	1.23	0.218	.6816636	5.357651
_cons	.3410684	.3080709	-1.19	0.234	.0580753	2.003048

Note: No difference in odds of mortality between the categories in adjusted analysis.

Supplementary Table S4. Results of multivariable analysis of the association between centre volume and mortality in-hospital.

ICD 9/10 Code	Description
81.01	Atlas-axis spinal fusion
0RG0	Fusion of occipital-cervical joint
0RG1	Fusion of cervical vertebral joint
0RG2	Fusion of two or more cervical vertebrae
1SA7	Fusion atlas and axis

Abbreviations: ICD, International Classification of Diseases.

Supplementary Table S5. International Classification of Diseases, 9th/10th revision, PCODEs corresponding to surgery for C2 fracture.
